# A rapid tick exposure test for monitoring acaricide resistance in *Rhipicephalus sanguineus* sensu lato ticks on dogs

**DOI:** 10.1186/s13071-024-06472-6

**Published:** 2024-09-28

**Authors:** Frans Jongejan, Laura Berger, Elias Papadopoulos, José Reck, Priscila Teixeira Ferreira, Fabio Barbour Scott, Barbara Rauta de Avelar, Brena Gava Guimarães, Thais Ribeiro Correia, Iris Hulsebos, Alita Petersen, Guilherme Klafke

**Affiliations:** 1 Department of Veterinary Tropical Diseases, Faculty of Veterinary Science, University of Pretoria, Private Bag X04, Onderstepoort, 0110 Republic of South Africa; 2 TBD International BV, BioScience Center, Runderweg 6, 8219 PK Lelystad, The Netherlands; 3 Laboratory of Parasitology and Parasitic Diseases, School of Veterinary Medicine, Aristotle University of Thessaloniki, University Campus, Thessaloniki, Greece; 4 Instituto de Pesquisas Veterinárias Desidério Finamor, Estrada Do Conde, 6000, Eldorado Do Sul, RS 92990-000 Brazil; 5 Instituto de Veterinária da Universidade Federal Rural Do Rio de Janeiro, Seropédica, RJ BR-465 Brazil

**Keywords:** Acaricide resistance, *Rhipicephalus sanguineus* ticks, Rapid Tick exposure Test, Dogs

## Abstract

**Background:**

Brown dog ticks (*Rhipicephalus sanguineus* sensu lato) are vectors of pathogens adversely affecting the health of dogs in many regions of the world. The three-host life cycle of *R. sanguineus* s.l., with all stages feeding on dogs, can lead to an uncontrolled build-up of large tick populations if not controlled by acaricides. However, frequent tick control on dogs using acaricides has led to the emergence of resistance to permethrin and fipronil. Currently, the larval packet test (LPT) is the standard tick resistance test, which is laborious, requires laboratory facilities, and takes at least 6 weeks before larvae derived from engorged female ticks can be tested. Our novel approach is to expose semi-engorged adult ticks to acaricides immediately after removing them from dogs, obtaining results within 24 h.

**Methods:**

Adult ticks from three laboratory colonies of *R. sanguineus* s.l. were tested in RaTexT^®^, a rapid tick exposure test in which ticks were confined to small compartments and exposed to an acaricide-impregnated, specially designed matrix. Resistance was confirmed by testing larvae derived from the same laboratory colonies using the LPT. RaTexT^®^ was also used to determine the susceptibility of *R. sanguineus* acaricides in dog shelters.

**Results:**

RaTexT^®^ detected resistance to permethrin in adult *R. sanguineus* s.l. ticks from two Brazilian laboratory colonies compared to a susceptible laboratory strain originating in Greece. Resistance was confirmed by LPT testing of larvae from the same colonies with resistance factors between 2.2 and 3.1. All laboratory strains were susceptible to fipronil. A suspected case of fipronil resistance at a dog shelter in Caxias do Sul, Brazil, was resolved within 24 h by testing adult ticks in RaTexT^®^ and could be attributed to improper treatment.

**Conclusions:**

RaTexT^®^ is a valuable tool for monitoring the development of resistance to synthetic pyrethroids or phenylpyrazoles in tick-infested dogs.

**Graphical Abstract:**

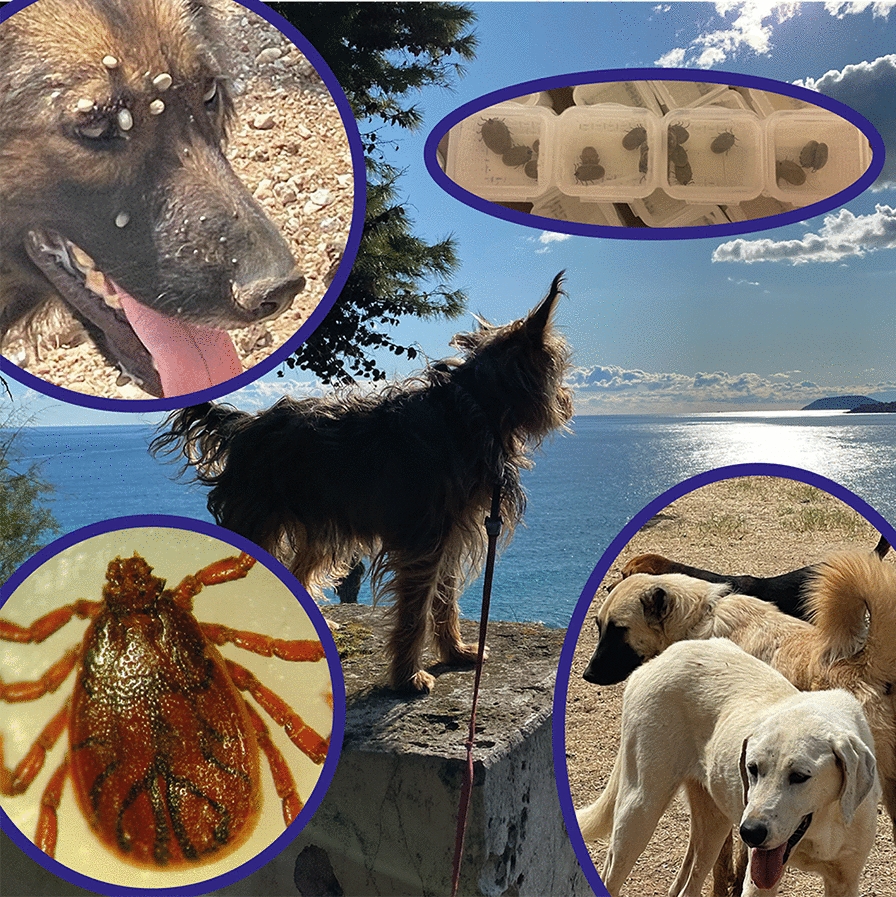

## Background

Ticks are among the most important vectors of diseases affecting dogs [1, 2]. Brown dog ticks (*Rhipicephalus sanguineus* sensu lato), particularly *Rhipicephalus sanguineus* and *Rhipicephalus linnaei*, are the most widely distributed tick species. Brown dog ticks are vectors of a range of bacterial and protozoan pathogens, causing canine ehrlichiosis and babesiosis, which adversely affect canine health worldwide [3]. With all stages feeding on dogs, their three-host life cycle can lead to an uncontrolled build-up of large tick populations [4, 5].

Tick control depends on applying acaricidal compounds to hosts [6]. In cattle, where ticks are repeatedly exposed to the same chemicals, selected highly resistant tick populations pose a significant global challenge to the livestock industry [7]. Plausible resistance mechanisms include target-site mutations and increased metabolic detoxification. Acaricide resistance of ticks on dogs is often anecdotal [8]. Although treatment failure may result from incorrect application of the acaricide, the persistence of ticks after correctly applied treatments indicates that resistance has developed [9]. Resistance can be confused with misuse of acaricides, because resistance tests are not readily available, and this is the rationale behind creating a rapid test that can be used in a clinic or kennel next to the dogs [8].

Resistance in *R. sanguineus* s.l. ticks has been reported against different acaricides, particularly organophosphates, macrocyclic lactones, synthetic pyrethroids and phenylpyrazoles. The first report of resistance to the cyclodiene compound dieldrin was in 1954 in *R. sanguineus* s.l. ticks, not cattle ticks [10]. The American dog tick, *Dermacentor variabilis*, has also been found resistant to dieldrin [11]. Furthermore, resistance to ivermectin, introduced in the mid-1980s, has also been reported in *R. sanguineus* s.l. ticks [12–15]. Reports of resistance against synthetic pyrethroids, sodium channel modulators that cause nerve excitation, have been documented in *R. sanguineus* ticks [13, 16, 17]. Finally, fipronil, a phenylpyrazole extensively used on dogs, has resulted in cases of resistance in dog ticks [13].

The Food and Agriculture Organization (FAO) recommends using the larval packet test (LPT) for resistance detection and monitoring in ticks [18]. The LPT employs a range of drug concentrations to cover 0–100% larval mortality [19]. Serial dilutions of acaricides are impregnated into filter papers in triplicate with a mixture of olive oil and trichloroethylene as the solvent. For running the tests, engorged female ticks must be collected, and their weight must be above a certain threshold for producing eggs. Proper guidance is required in collecting, handling and submitting engorged ticks to diagnostic laboratories. For most tick species, incubation conditions for engorged female ticks for eggs and larval production should be 27 °C and 85% relative humidity. The main drawbacks of the LPT are that the test is laborious, requires laboratory facilities and trained personnel, and takes at least 6 weeks before testing with larvae derived from engorged female ticks collected from animals in the field can be completed. Moreover, it demands a significant number of fully engorged female ticks collected simultaneously from the host, which may be difficult for companion animals.

Recently, we introduced RaTexT^®^, a rapid tick exposure test, to detect acaricide resistance in livestock ticks (F. Jongejan, unpublished information). The novel rapid test is based on the principle that free-living unfed ticks collected from the environment or partly engorged ticks recently removed from their hosts can be utilised to detect resistance by exposing them to lethal concentrations of an acaricidal compound. During their movement on the impregnated matrix, ticks are exposed to the acaricides, mimicking the behaviour of ticks that embark on an acaricide-treated host. Moreover, the test is also based on the resistance intensity protocol from the latest World Health Association (WHO) guidelines for resistance detection in malaria mosquito vectors, which proposed evaluation under 1×, 5× and 10× drug concentrations, revealing a low, moderate and high resistance intensity, respectively [20, 21]. This paper evaluates a novel rapid tick exposure test for detecting acaricide resistance in *R. sanguineus* ticks collected from dogs.

## Methods

### Tick strains

A susceptible reference colony of *R. sanguineus* s.l. (designated as CHRYSO) originated from a dog shelter in Chryso (41°03′29″N 23°39′00″E), northeastern Greece. The colony was initiated in 2022 and regularly replenished with freshly collected ticks from the same kennel. The ticks were specific-pathogen-free, particularly *Ehrlichia canis* and *Babesia vogeli*. Moreover, this strain was cultivated after the owners indicated that they were not using any systematic tick control. Subsequently, larvae derived from engorged ticks collected from their premises were tested by LPT to confirm that these ticks were susceptible to synthetic pyrethroids and fipronil, justifying their use as a susceptible reference strain in this study.

Two colonies of *R. sanguineus* s.l. ticks were collected in Brazil and maintained at the Departamento de Parasitologia Animal of Universidade Federal Rural do Rio de Janeiro (UFRRJ) facilities in Seropédica, RJ, Brazil. These were designated as UFRRJ and RSPA, and adult and larval ticks were used for the experiments. All colony ticks were maintained on rabbits under controlled laboratory conditions as described previously [22]. Furthermore, a field population of *R. sanguineus* s.l. ticks were collected from a dog shelter at Caxias do Sul, Rio Grande do Sul, Brazil (designated as CAXIAS strain) and tested in RaTexT^®^ without setting up a laboratory colony.

### Construction of the RaTexT^®^

The RaTexT^®^ was conducted in a transparent polypropylene container with seven rows of four small, interconnected compartments, where adult ticks are exposed to an acaricide-impregnated nylon matrix immediately after being removed from dogs. Each compartment was identified by a label, indicating control, 1×, 5× or 10×. Each row of interconnected compartments was also marked with either permethrin or fipronil. The nylon matrix, obtained from Cerex Advanced Fabrics, was cut into the required sizes to fit into a compartment of 2 × 2 cm using a ScanNCut SDX900 (Brother Sewing Machines). Both acaricides were diluted in olive oil and acetone (1:2), the latter replacing trichloroethylene for safety reasons. Concentrations of permethrin (Advantix, Elanco Animal Health, Monheim, Germany) in the 1× recommended dose (RD), 5× concentrated dose (CD) and 10× concentrated dose (CD) were 1.5 mg/ml, 7.5 mg/ml and 15 mg/ml, respectively. Concentrations of fipronil (Boehringer Ingelheim, Ingelheim am Rhein, Germany) in the 1× RD, 5× CD and 10× CD were 1.0 mg/ml, 5.0 mg/ml and 10 mg/ml, respectively. Oil-based food colouring was added to distinguish between the different matrices as follows: control (white), 1× RD (green), 5× CD (orange) and 10× CD (red).

The matrix pieces were impregnated with diluted acaricides inside separate plastic zip bags using 0.37 ml per piece. Each acaricide was adequately mixed with the matrix pieces and left inside the zip bag for 30 min to ensure a homogeneous coating. Afterwards, the matrix pieces were transferred to an aluminium tray and thoroughly dried in a fume hood overnight. Finally, each piece of the matrix was fixed to the bottom of a compartment of the RaTexT^®^ container with a small drop of Wacker Elastosil E4 glue. The inside of the lid remained uncovered.

### The RaTexT^®^

Free-living adult ticks were collected from the shelter walls and floors for the CAXIAS field strain. Fed *R. sanguineus* s.l. ticks were manually removed from dogs and collected into a 100 ml plastic beaker, which was subsequently emptied into a shallow carton box (30 × 22 × 3 cm), where they were allowed to move around and were selected based on sex, vitality (actively walking) and preferred size. Five to eight ticks were inserted into each compartment of the RaTexT^®^ box to perform the test, starting with the control and ending with the highest acaricide concentration (10×). After that, all lids were firmly closed, and the RaTexT^®^ container was placed inside a plastic zip bag with a piece of moist tissue and sealed completely. Each container was left at room temperature and out of direct sunlight for 24 h. After 24 h, ticks were removed from the compartments, starting with the controls, to record the number of live and dead ticks. A tick was considered dead or ‘knocked down’ if it had lost its ability to walk. This procedure was facilitated by placing each tick inside a small grey circle of 0.8 mm diameter on the data capture form. Ticks that could walk out of the circle were recorded as alive versus those that could not move even after stimulating them with breath and gently prodding them with blunt forceps. After counting, all ticks were discarded inside a plastic zip bag containing ethanol, and forceps were cleaned with ethanol after use.

### Larval packet test (LPT)

The LPT was carried out as described previously [18]. Briefly, serial dilutions of permethrin (Advantix, Elanco Animal Health) (0.004–15 mg/ml) and fipronil (Frontline, Boehringer Ingelheim) (0.000256–4 mg/ml) were impregnated into 7 × 10 cm Whatman quality (no. 1) filter papers (Merck Life Science, Amsterdam) in triplicate using a mixture of olive oil and acetone as the solvent. The control filter papers were impregnated only with acetone and olive oil (2:1). Each filter paper was folded in width to create a packet, sealed by bulldog clips [18]. Using the paintbrush, a small cluster containing approximately 100 larvae was collected from the rim of an open tick rearing tube and transferred into the packets.

The packets were incubated at room temperature for 24 h in a desiccator with 85% relative humidity. After that, live and dead larvae were counted, starting with the control packets.

### Statistical analysis

Abbott’s formula was used to correct for mortality of 10% or less:

$$ Corrected\,mortality\,percentage\, = \,\frac{\% \,test\,mortality\, - \,\% \,control\,mortality}{{100\, - \,\% \,control\,mortality}}\, \cdot \,100. $$

A 90% mortality cut-off was used based on the World Association for the Advancement of Veterinary Parasitology (WAAVP) guidelines recommendation for acaricide efficacy in the field [45]. A decision tree was used to determine whether ticks were susceptible or carried a low, medium or high resistance level (Fig. 3). For the LPT, a probit analysis was performed on the mortality results using PoloPlus (version 1.0, LeOra Software) software to generate the dose–response plots. The following parameters were determined for each test: lethal concentrations for 50% (LC_50_), 95% confidence intervals (95% CI), and the slope of the regression line. Resistance ratios (RR) and their 95% CIs were generated with PoloPlus software using the following formula described by Robertson et al. [23].

$$ Resistance\,ratio\, = \,\frac{ LC50\,of\,the\,resistant\,tick\,strain}{{ LC50\,of\,the\,susceptible\,tick\,strain}}. $$

## Results and discussion

**Fig. 1:**
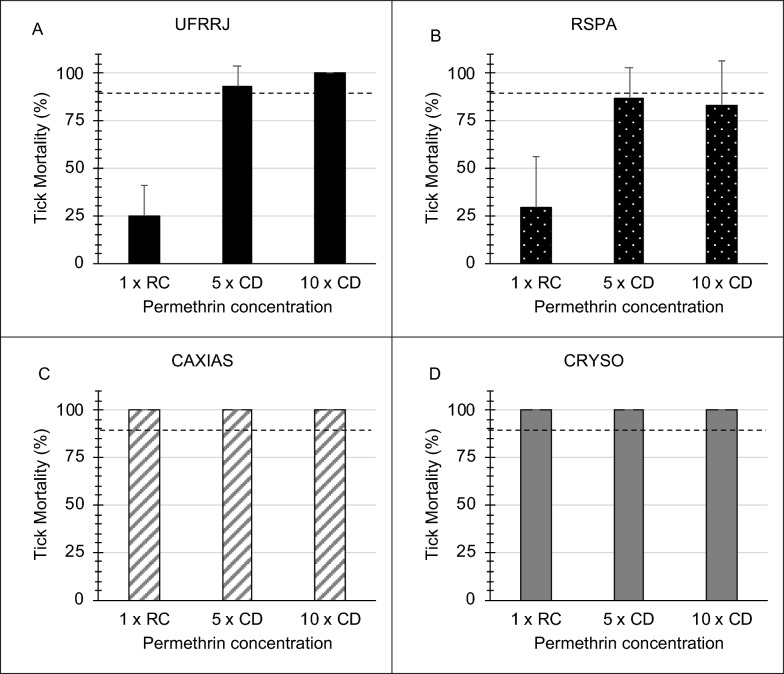
RaTexT^®^ against permethrin with *Rhipicephalus sanguineus* s.l.-resistant strains UFRRJ (**A**) and RSPA (**B**) and susceptible field populations CAXIAS (**C**) and CHRYSO (**D**). *RC* recommended concentration, *CD* concentrated dose. Error bars represent the standard deviations. The dashed line represents the 90% mortality cut-off value

Ticks from *R. sanguineus* s.l. colonies and the field isolate were confined to the RaTexT^®^ compartments and exposed to the 1× recommended concentration and 5× and 10× concentrated doses of a commercial formulation of permethrin. The results of the rapid test with permethrin are presented in Fig. 1. For the UFRRJ strain (Fig. 1A), the mean mortality for the 1×, 5× and 10× doses was 25%, 93.3% and 100%, respectively. RaTexT^®^ identified a low level of resistance to permethrin according to a decision tree with a cut-off of 90% mortality (Fig. 3). The RSPA strain showed a high level of resistance, with mean mortality of 28.9%, 86.7% and 83.3% at the 1×, 5× and 10× doses, respectively (Fig. 1B). The CAXIAS field isolate collected from the dog shelter in Brazil (Fig. 1C) and CHRYSO strain from Greece (Fig. 1D) were susceptible to permethrin, with 100% mortality at all doses. The controls carried little, if any, mortality, which was corrected using Abbott’s formula if required.

**Fig. 2:**
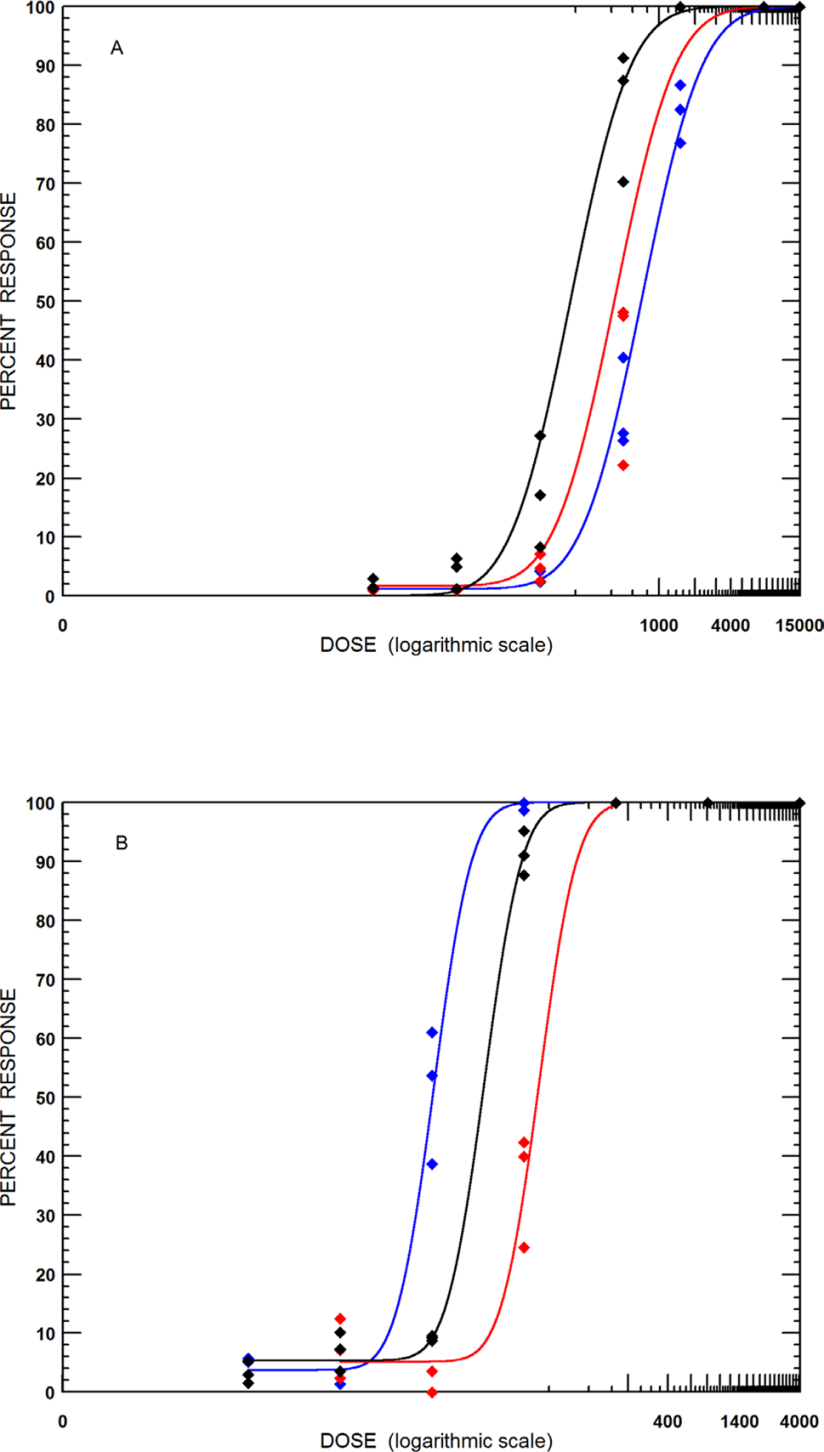
Comparison of strains of *Rhipicephalus sanguineus* s.l. larvae with the larval packet test (LPT) against permethrin (**A**) and fipronil (**B**). RSPA isolate (blue line and dots), UFRRJ colony (red line and dots). CHRYSO field population (black line and dots). Doses in parts per million

Engorged female ticks from UFRRJ, RSPA and CHRYSO strains were further utilised to obtain larvae tested in the LPT. These results are presented in Fig. 2A and Table 1. The LC_50_ for permethrin in the CHRYSO strain, 0.239 mg/ml, was significantly lower than the other two colonies, which presented resistance ratios of 2.2-fold and 3.1-fold for UFRRJ and RSPA, respectively. Both Brazilian colonies (UFRRJ and RSPA) were isolated from field-collected ticks in 2009 and 2023 in areas where they could have been previously exposed to synthetic pyrethroids. Even without selection after isolation, ticks could maintain their pyrethroid-resistant status for several generations, as shown previously for *Rhipicephalus microplus* ticks [24]. The tick strain from the Greek kennel was initiated after the owners indicated they were not using systematic tick control. Subsequently, testing of larvae in the LPT confirmed that these ticks were susceptible to synthetic pyrethroids and fipronil, justifying their use as a susceptible reference strain in this study.

**Table 1:** Results of the larval packet test with three strains of *Rhipicephalus sanguineus* larvae against permethrin

Acaricide	Strain	*N*	Slope (SE)	LC_50_ (95% CI) mg/ml	RR
Permethrin	UFRRJ	2189	3.634 (0.112)	0.524 (0.451–0.596) a	2.192
	RSPA	1913	2.964 (0.269)	0.734 (0.631–0.834) b	3.071
	CHRYSO	1318	3.154 (0.247)	0.239 (0.179–0.305) c	–

*N* number of larvae, *SE* standard error, *LC*_*50*_ median lethal concentration, *95% CI* 95% confidence interval, *RR* resistance ratio; a, b, c: different letters represent significantly different LC_50_ values, based on the non-overlapping of 95% CIs

For fipronil, all the strains were considered susceptible. The mortality of all ticks was 100% at all doses tested in the RaTexT^®^ boxes. When tested with the LPT (Fig. 2B; Table 2), the UFRRJ strain showed the highest LC_50_ (0.029 mg/ml) compared to the other strains (RSPA = 0.007 mg/ml; CHRYSO = 0.015 mg/ml). Nevertheless, clinical efficacy trials previously demonstrated the UFRRJ strain's susceptibility to fipronil, with 100% efficacy for 42 days in dogs artificially infested with the UFRRJ strain (data not shown).

**Table 2:** Results of the larval packet test with three strains of *Rhipicephalus sanguineus* larvae against fipronil

Acaricide	Strain	*N*	Slope (SE)	LC_50_ (95% CI) mg/ml	RR
Fipronil	UFRRJ	1526	1.646 (0.114)	0.029 (0.022–0.035) a	1.933
	RSPA	1614	3.935 (0.472)	0.007 (0.006–0.008) b	0.466
	CHRYSO	1289	4.405 (0.433)	0.015 (0.013–0.018) c	–

*N* number of larvae, *SE* standard error, *LC50* median lethal concentration, *95% CI* 95% confidence interval, *RR* resistance ratio; a, b, c: different letters represent significantly different LC50 values, based on the non-overlapping of 95% CIs

The CAXIAS strain was collected in a dog shelter where the personnel suspected resistance to fipronil. It was alleged that treatments were frequent (every week), and infestation was uncontrolled. The investigation on the premises confirmed the abundant presence of ticks in some dog kennels. It was found that the treatments were made with a fipronil pour-on formulation marketed for cattle. In this case, the results obtained with the rapid test indicated that resistance to fipronil was not the case. Still, irregular tick control management was employed with off-label use of bovine formulations in dogs. Collection of engorged female ticks in the Caxias shelter to test subsequent larvae with the LPT was not possible. Nevertheless, the rapid test was efficient in showing that efficacy failure was not due to resistance.

Resistance of *R. sanguineus* s.l. ticks to synthetic pyrethroids has been reported from different parts of the world [25], first from Panama in 2001 [26] and more recently from India [16, 27] and West Africa [17]. The first documentation of *R. sanguineus* s.l. permethrin resistance in 2015 in the United States [28] has been followed up by further evidence in other populations originating from Florida and California [29]. Resistance to pyrethroids has been reported to be associated with mutations in the sodium channel gene, resulting in phenotypic resistance profiles [30].

**Fig. 3:**
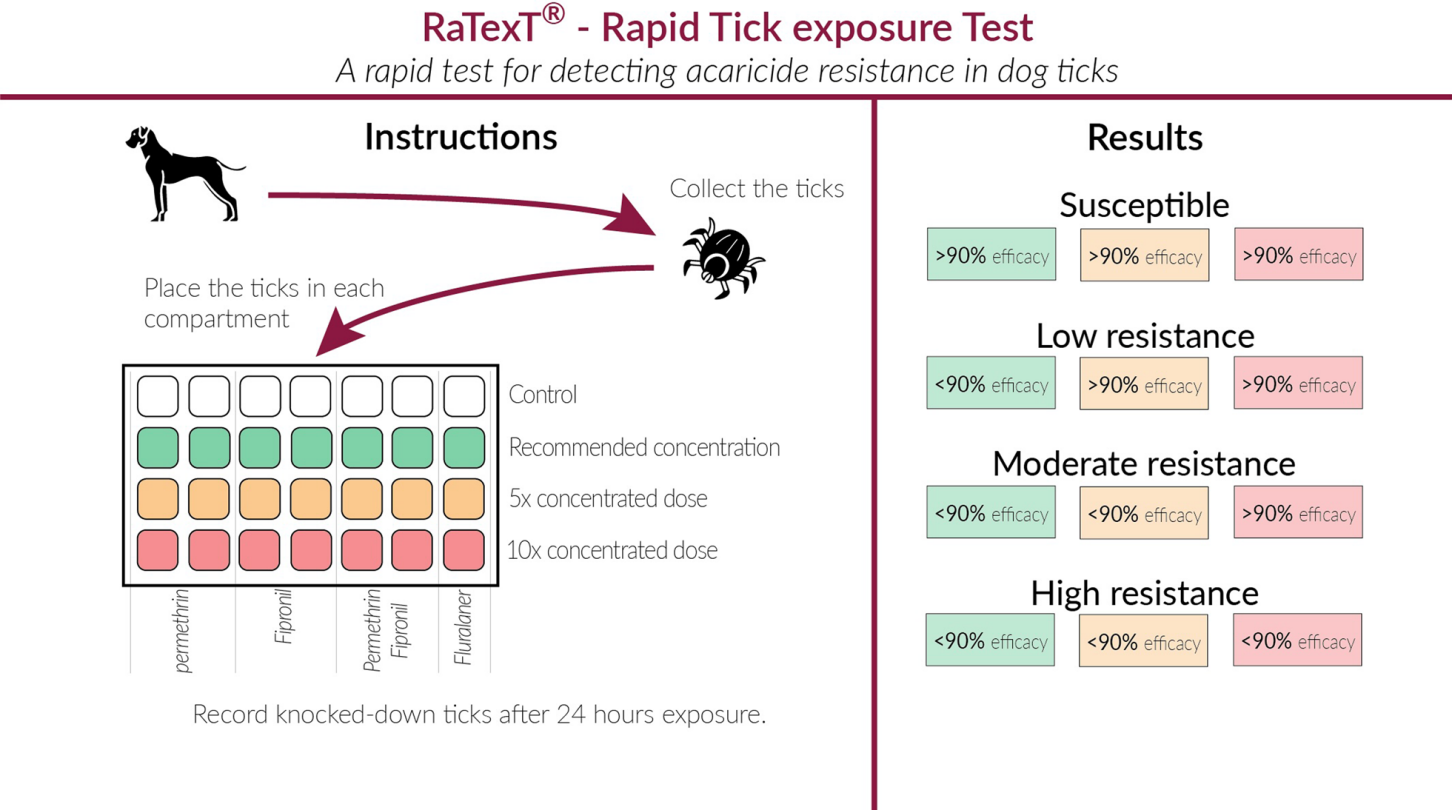
Rapid Tick exposure Test (RaTexT) outline for acaricide resistance monitoring in dog ticks

RaTexT^®^ for dog ticks has the potential to become a valuable tool for clinical practice and resistance surveillance programs by providing results within hours. In contrast, the LPT takes at least 6 weeks. It is following the tendency to increase the availability of point-of-care testing (POCT) in small animal clinics [31]. The final design of the RaTexT^®^ box for resistance surveillance of dog ticks contains compartments with permethrin or fipronil (Fig. 3). Combination products of fipronil and permethrin are also on the market and have been demonstrated to be highly effective against *R. sanguineus* s.l. [32] and in preventing the transmission of *Babesia canis* to dogs by *Dermacentor reticulatus* and *E. canis* by *R. sanguineus* s.l. [33]. Hence, the combination of permethrin and fipronil is included in the final design as well (Fig. 3). Finally, RaTexT^®^ is also charged with isoxazoline and fluralaner, creating the opportunity to simultaneously screen ticks for their susceptibility to different classes of acaricides in kennels with a significant number of ticks on dogs.

## Data Availability

RaTexT^®^ is a registered trademark and has been patented under registration number 2036918. All data supporting the conclusions of this study are included in the manuscript.

## Abbreviations

LPT: Larval packet test
RaTexT: Rapid Tick exposure Test
